# Characterization of loss of chromosome Y in peripheral blood cells in male Han Chinese patients with schizophrenia

**DOI:** 10.1186/s12888-023-04929-z

**Published:** 2023-06-27

**Authors:** Lanrui Jiang, Mengyuan Song, Feng Song, Yuxiang Zhou, Hewen Yao, Gangqin Li, Haibo Luo

**Affiliations:** 1 Department of Forensic Genetics, West China School of Basic Medical Sciences & Forensic Medicine, Sichuan University, Chengdu, 610041 Sichuan Province China; 2 Department of Laboratory Medicine, West China Hospital, Sichuan University, Chengdu, Sichuan China; 3 Department of Forensic Psychiatry, West China School of Basic Medical Sciences & Forensic Medicine, Sichuan University, Chengdu, 610041 Sichuan Province China

**Keywords:** Loss of chromosome Y (LOY), Schizophrenia (SCZ), Droplet digital PCR (ddPCR), Age

## Abstract

**Background:**

Schizophrenia (SCZ) has a global prevalence of 1% and increases the risk of mortality, reducing life expectancy. There is growing evidence that the risk of this disorder is higher in males than in females and it tends to develop in early adulthood. The Y chromosome is thought to be involved in biological processes other than sex determination and spermatogenesis. Studies have shown that loss of chromosome Y (LOY) in peripheral blood cells is associated with a variety of diseases (including cancer) and increased all-cause mortality. An analysis of the relationship between LOY and schizophrenia is warranted.

**Methods:**

A total of 442 Chinese males (271 patients with schizophrenia vs. 171 controls) were included in this study. The copy numbers of the Y and X chromosomes were detected by positive droplets targeting the amelogenin gene (AMEL) on the Y chromosome and X chromosome (AMELY and AMELX, respectively), using droplet digital PCR (ddPCR). The LOY percentage was defined as the difference between the concentration of AMELX and the concentration of AMELY divided by the concentration of AMELX, denoted as (X - Y)/X.

**Results:**

In the Han Chinese population, the LOY percentage was higher in the schizophrenia group than in the control group (*p* < 0.05), although there was no significant difference in the presence of LOY between the two groups. A strong correlation was found between the average of the disease duration and the average of the LOY percentage (R^2^ = 0.506, *p* = 0.032). The logistic regression analysis implied that the risk of LOY increases by 0.058 and 0.057 per year according to age at onset and duration of disease, respectively (*p*_onset_ = 0.013, *p*_duration_ = 0.017).

**Conclusions:**

In the Han Chinese population, the LOY percentage of the disease group was significantly different from that of the control group. The age of onset and duration of schizophrenia might be risk factors for LOY in peripheral blood cells. A larger sample size and expanded clinical information are needed for more in-depth and specific analyses.

**Supplementary Information:**

The online version contains supplementary material available at 10.1186/s12888-023-04929-z.

## Background

The term schizophrenia (SCZ) was first used by Swiss psychiatrist Eugen Bleuler [1] in the 19th century to refer to a complex mental disorder with far-reaching effects on both individuals and society. The global prevalence of SCZ is approximately 1%, which results in a sizable schizophrenia population in many large countries, such as the United States [2], imposing a large burden on health care and society each year. Currently, the diagnosis of this psychiatric disorder relies on clinical assessment, mainly based on the medical history and mental status examination. The characteristic symptoms of SCZ include positive symptoms (delusions, hallucinations), negative symptoms (reduced volition, emotional indifference), and cognitive dysfunction. Attention should also be given to the differential diagnosis of SCZ and other psychiatric disorders, such as bipolar disorders. In 1973, a study by the World Health Organization (WHO) was carried out with 811 participants to derive a system of 12 signs and symptoms for the identification of schizophrenia [3]. Nevertheless, an analysis by John McGrath et al. [4] showed that the incidence may vary with migrant status, urbanity, economic status, and other factors. There is a large impact of this disorder on life expectancy [5], with an increased risk of suicide and mortality in patients with SCZ compared to normal individuals.

For decades, researchers have generally accepted the neurodevelopmental hypothesis to explain the impact of additional environmental factors on the incidence of SCZ, including maternal infections, intrauterine growth retardation, and complications of pregnancy and childbirth [6]. In addition, the interaction of genetic and environmental factors is expected to increase the risk of the disease, resulting in heterogeneity among patients. A growing body of evidence strongly indicates that heritability is a crucial factor in the development of SCZ. In a genome-wide association study [7], 108 physically distinct loci were correlated with SCZ, and the number of associated loci has increased with additional large-scale sequencing studies [8, 9]. Moreover, advances in sequencing technology have allowed many researchers to identify multiple rare copy number variants (CNVs) significantly associated with the risk of SCZ, enhancing understanding of the genetic architecture of SCZ [10–12]. However, the overlap of risk factors and underlying mechanisms leads to the possibility that many of the genetic variants associated with SCZ may also be associated with other psychiatric disorders. New genetic biomarkers with diagnostic value are needed.

Recently, James B et al. [13] and Hannah E Jongsma et al. [14] have suggested that males are at higher risk of the disorder and are more likely to develop it in early adulthood [13, 15]. Since the prevalence is higher in men, it is interesting to explore male-specific alterations. It is well known that males and females differ in longevity. As previous studies have shown, in species in which sex is determined by the presence of the Y chromosome, females (with XX chromosomes) consistently have a longer lifespan [16, 17]. The Y chromosome is specific to males, and its size (as one of the shortest chromosomes in the human karyotype), it is nevertheless crucial for correct male development [18]. Loss of chromosome Y (LOY) is one of the most common somatic mutations that can occur at any stage of a male individual’s life, and its prevalence increases with age. An increasing number of studies have revealed that LOY may be associated with a variety of health issues, such as cancer, cardiovascular disease, age-related disorders, and smoking status [19–21]. In recent years, methods for the detection of LOY have been updated and refined, including traditional fluorescent in situ hybridization (FISH) [22], as well as emerging Illumina Single Nucleotide Polymorphism (SNP) arrays, next-generation sequencing technologies [19, 21], qPCR [23], multiplex fluorescent PCR [24], and the first absolute quantification of LOY percentage using droplet digital PCR (ddPCR), developed by Danielsson et al. [25]. These methods provide the technical basis and foundation for future studies.

Notably, one study found that the frequency of LOY in peripheral blood cells was associated with suicide completion [26]. It has been reported that suicide rates are higher in men than in women in most countries [26–28], and genetic factors, particularly sex chromosomes, are thought to partially explain the sex differences in outcomes of mental illness and various psychological problems [29]. This suggests that the high prevalence of SCZ in males may be associated with LOY, which is the question we explore in the present study. Takashi Hirata’s team exploried this issue in the Japanese population, but they found no significant difference in the frequency of LOY between SCZ patients and controls [30]. However, this research still motivated our current study, as it remains unclear whether there are differences in LOY between individuals with schizophrenia and normal individuals in the Han Chinese population. As there is a lack of biomarkers to diagnose SCZ in clinical practice, an association between LOY and SCZ will enhance the ability to diagnose SCZ using biomarkers.

## Methods

### Samples

The study has been carried out in accordance with The Code of Ethics of the World Medical Association (Declaration of Helsinki) and the samples were obtained with the approval of the Medical Ethics Committee of Sichuan University (reference number: K2018092). The blood samples were obtained from participants among the Han Chinese population. The written informed consent and related information were provided. This study cohort consisted of 271 males with SCZ (median age: 48 years old, Figure S1). Moreover, to conduct a more comprehensive analysis, we also used the data from 171 Han Chinese normal males (median age: 44) as the control group (in another accepted manuscript, in-press) for comparison. The related clinical characteristics of the patients are presented in Table 1(detailed data are shown in the supplementary materials).

**Table 1:** Demographic and clinical characteristics of participants for loss of chromosome Y study

	Schizophrenia (n = 271)	Control (n = 171)	*P*-value
Age (years) (median [IQR])	48 [ 42, 56]	44 [23, 66]	0.100*
Age of onset (years)(median [IQR])^a^	31 [23.25, 41]	—	—
Duration of SCZ (years)(median [IQR])^a^	13 [8, 23]	—	—
Presence of LOY^b^	37 / 271	27 / 171	0.534**
Symptoms^c^ (known / unknown)	258 / 13	—	—
Negative symptom	64		
Positive symptom	218		
Residual symptoms	245 / 271	—	—
Medication (Clozapine:mg/day)(median [IQR])^d^	150 [75, 281.25]	—	—
Effect of therapy (Ineffective / partially effective / effective / unknown)	1 / 223 / 33 / 14	—	—
Side effect (without / EPS / Leukocytopenia / constipation / unknown)	244 / 1 / 11 / 1 / 14	—	—
Sleep disorder (with / without / unknown)	14 / 245 / 12	—	—
Violence risk rating (0 / 1 / 2 / 3 / 4 / 5 / unknown)	127 / 4 / 3 / 5 / 10 / 11 / 111	—	—
Smoking (yes / no / unknown)	7 / 209 / 55	—	—

**Notes**:a). Information on age at onset and duration of illness was collected for 186 out of 271 patients with SCZ; b). we evaluated the LOY percentage > 0.1 as LOY; c). certain patients have both positive and negative symptoms; d). 111 of 271 patients were on different doses of clozapine; *Mann–Whitney U-test; **Pearson Chi-Square.

**Abbreviations**: IQR, interquartile range; SCZ, schizophrenia; LOY, loss of chromosome Y; EPS, extra pyramidal symptoms.

### Determination of LOY in peripheral blood

Genomic DNA was extracted using the salting out method or QIAamp DNA Mini Kit (Qiagen, Hilden, Germany) in accordance with the manufacturer’s recommendations and was quantified by Nanodrop 1000 (Thermo Fisher Scientific, Waltham, USA). The DNA samples were stored at -20 ℃ before use.

Quantification of LOY was conducted by TaqMan assay based on the homologous Amelogenin genes (AMEL) located on both the X and Y chromosome that differ by 6 bp in length, which could be amplified with the same primers. The TaqMan probes targeting AMELX and AMELY, respectively, were designed to quantify the copies of X and Y chromosomes without amplification bias [25]. TaqMan primers and probes were ordered from Thermo Fisher Scientific (MA, USA) with article number C_990000001_10. DNA samples with concentrations beyond 20 ng/µl were digested with HindIII (Thermo Fisher Scientific MA, USA) at 37℃ for 15 min. Then, a 50ng digested and diluted DNA sample was used as input DNA in ddPCR™ Supermix for Probes (No dUTP) (Bio-Rad Laboratories, Inc., CA, USA) together with TaqMan probes and primers.

Droplets were generated by the QX200 Droplet Generator (Bio-Rad Laboratories, Inc., CA, USA) following the manufacturer’s instructions. The target and background DNA were randomly distributed among the droplets. Subsequently, the droplets were amplified on the C1000 Touch Thermal Cycler (Bio-Rad Laboratories, Inc., CA, USA) using the following conditions provided in the manufacturer’s instruction: 95℃ for 10 min, 40 cycles of 94℃ for 30 s and 60℃ for 1 min, ended with 98℃ for 10 min and a 10℃ hold. The amplification-completed 96-well plate was then transferred into the QX200 Droplet Reader (Bio-Rad Laboratories, Inc., CA, USA). The fluorescences of the droplets were read and analyzed one by one by using Bio-Rad’s software QuantaSoft (version 1.7), where FAM was targeted to AMELY and shown in blue while VIC was targeted to AMELX and shown in green. Each droplet went through a two-color optical detection system as a separate unit. All samples were run in duplicates and the standard deviation of the measured ratios was calculated. If the standard deviation was 1.2 or higher, the samples were reanalyzed according to the criteria of the previous study.

LOY percentage by ddPCR was calculated as follows: LOY percentage = (the concentration of AMELX - the concentration of AMELY)/the concentration of AMELX. We evaluated the presence or absence of LOY, coding LOY percentage > 0.1 as LOY and the LOY percentage ≤ 0.1 as normal. The threshold was based on the findings of previous studies [31, 32].

### Statistical analysis

To explore the correlation between factors such as age and LOY and whether there are differences between groups, Pearson correlation, Mann–Whitney U-test, Pearson Chi-Square as well as logistic regression analysis were performed by SPSS software (version 20, IBM Corporation, Armonk, NY, USA), as appropriate. Statistical significance was adopted as two-tailed *p*-values < 0.05. Moreover, visualization analysis was conducted by Hiplot tool online (https://hiplot.com.cn/home/index.html).

## Results

**Fig. 1:**
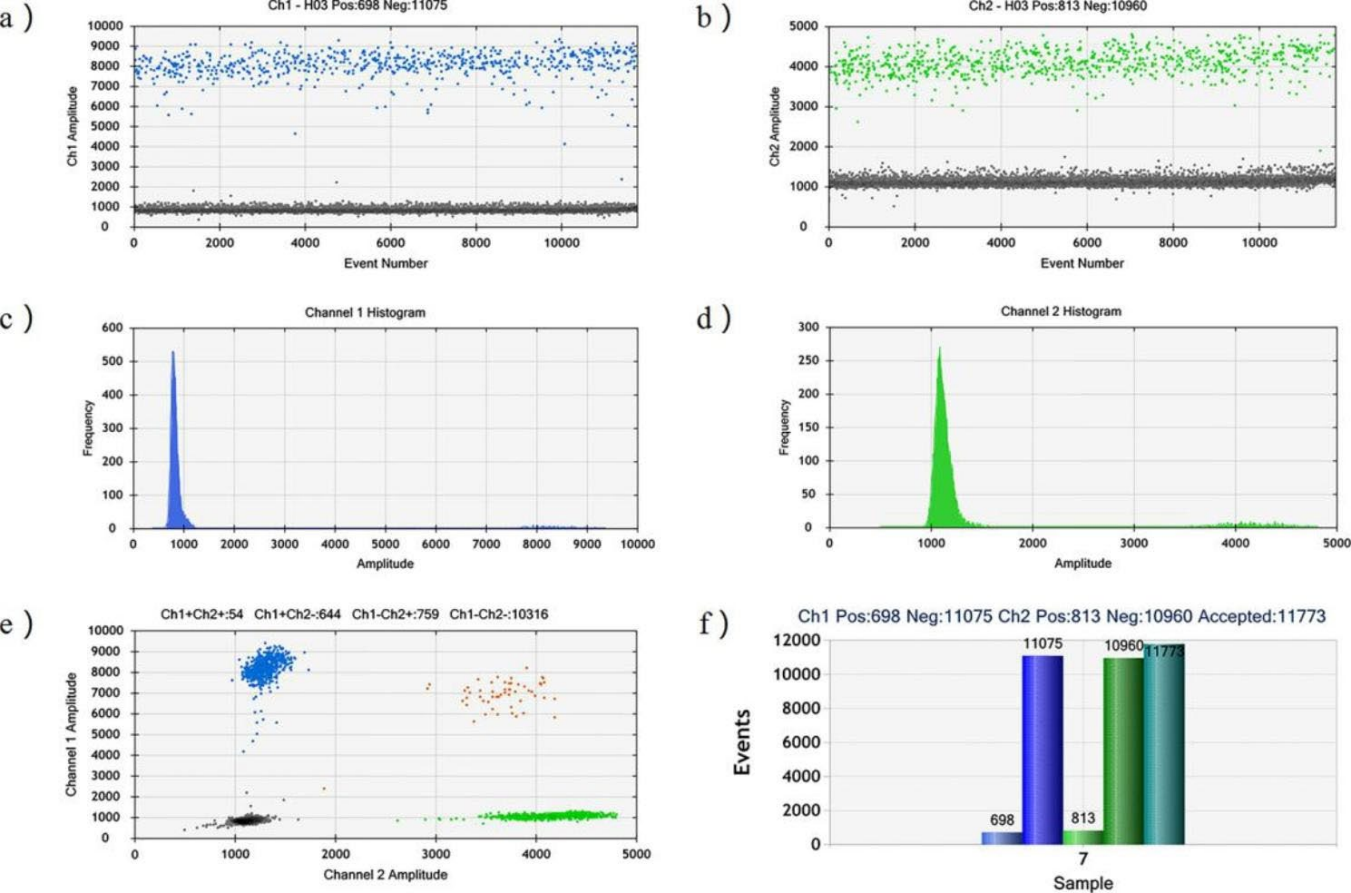
An example ddPCR plot. **(a)** shows the FAM channel that targeted AMELY in blue color while **(b)** shows the VIC channel targeted AMELX in green. The y-axis in (a) and (b) represented the amplitude of the fluorescence. Grey dots were negative droplets without gene copy. **(c)** and **(d)** indicate the frequency distribution of fluorescence signals in the FAM and VIC channels, respectively. **(e)** is a two-dimensional presentation of the droplets for both channels. **(f)** is a histogram of the number of positive and negative events for the two channels

The dots in Fig. 1a-b reflect the droplets detected in the FAM channel and VIC channel, with the grey dots indicating negative droplets, the blue dots reflecting AMELY, and the green dots corresponding to AMELX. Figure 1c-d is a graphical representation of the data in Fig. 1a-b, reflecting the frequency distribution of the fluorescence signals of the two channels. Figure 1e provides a two-dimensional image that combines the two channels. Figure 1f is a histogram of the number of positive and negative droplets detected in the sample in the two channels targeting AMELX and AMELY. Finally, the software automatically derived the corresponding gene copy numbers based on the number of positive and negative droplets and the Poisson distribution principle.

### Correlations between age and LOY in the blood of SCZ patients and healthy controls

**Fig. 2:**
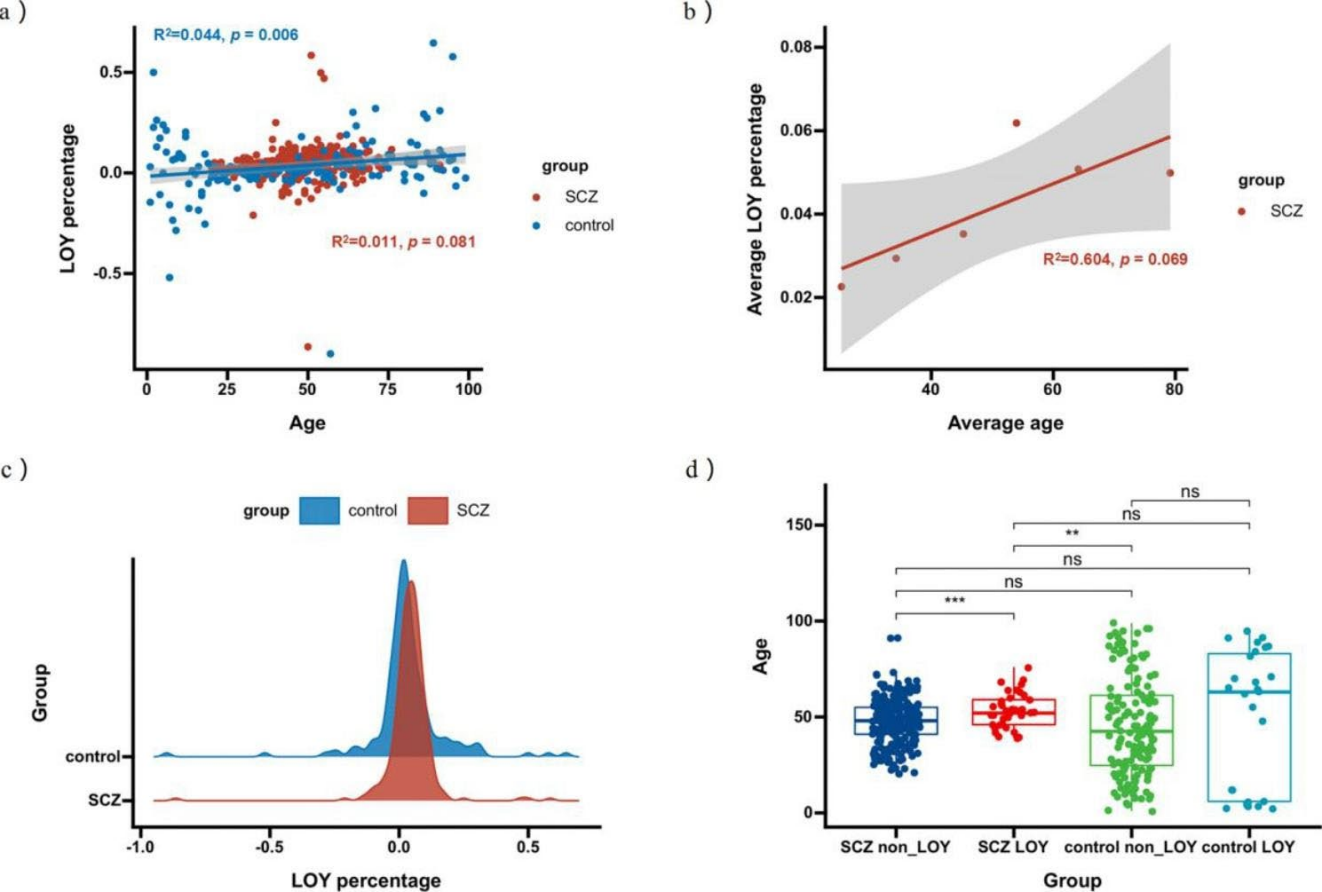
**(a)** is a scatter plot between the age of the samples and LOY percentage in the SCZ group (in red) and the control group (in blue). The x-axis is the age of the participants and the y-axis is their LOY percentage. The correlation index R^2^ was 0.011 (*p* = 0.081) in the schizophrenic group while the R^2^ equals 0.044 (*p* = 0.006) in controls. **(b)** shows reanalysis results of the correlation between the average age and the corresponding average ratio for each age group in the SCZ patients. **(c)** reflects the distribution of the overall LOY percentage for the SCZ group (red) and the control group (blue), with the height representing the frequency of ratio occurrence. **(d)** presents the difference between the ages of schizophrenic patients and healthy males with and without LOY.

Pearson correlation analysis of age and the LOY percentage was performed and revealed a weak positive correlation in the SCZ group (Fig. 2a). Specifically, the correlation coefficient was 0.106 (R^2^ = 0.011) with a *p* value of 0.081 in the SCZ group, while the coefficient was 0.210 (R^2^ = 0.044) with a *p* value of 0.006 in the control group. The LOY percentage distributions for the two groups are shown in Fig. 2c; the data of the SCZ group are shifted to the right compared to those of the control group. This implies that the SCZ group may be more likely to have a higher percentage of Y chromosome loss. We also calculated the mean LOY percentage in each age group (grouped by the average age). The correlation coefficient increased to 0.777, but the correlation was not statistically significant (R^2^ = 0.604, *p* = 0.069) (Fig. 2b).

The results showed that 13.653% of males with SCZ and 15.789% of healthy males exhibited LOY, and there was no significant difference in the prevalence of LOY between the two groups (*p* = 0.534). We found a significant difference in age between the SCZ patients with and without LOY (*p* = 0.003). In the control group, there was no significant difference in age between the individuals with LOY and those without LOY (Fig. 2d). The results suggest that age might increase susceptibility to LOY in schizophrenia patients.

**Fig. 3:**
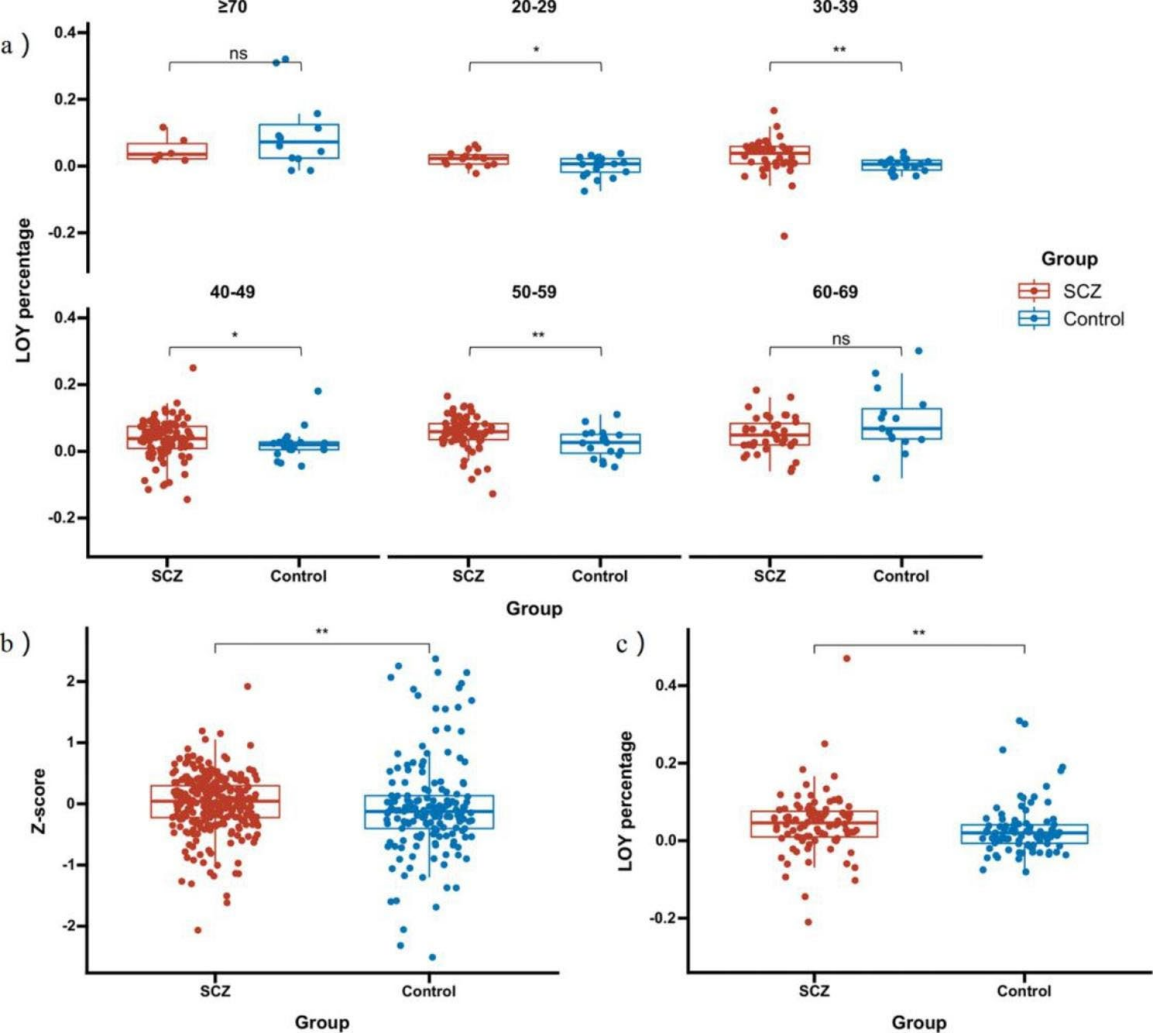
**(a)** Intergroup comparisons of the LOY percentage for each age range. Significant differences were found in the 20–29 years old group, 30–39 years old group, 40–49 years old group, and 50–59 years old group. The ratio data was slightly higher in SCZ patients than in the control group. **(b)** The LOY percentage were age-corrected by the residual method, and the calculated Z-score was used for comparison between groups. A significant difference was found between the two groups (*p* < 0.05). **(c)** Age matching and screening of 184 samples by Propensity Score Matching method showed that the percentages of SCZ patients were higher than that of controls with statistical significance

Since we found that the LOY percentage changed with age, which is consistent with previous studies, we adjusted for the effect of age by various approaches, including age groups, the residual correction method and propensity score matching (PSM). After correcting for age, we found a significant difference in the LOY percentage between SCZ patients and healthy controls (*p* < 0.05, Fig. 3). The results suggest that the LOY percentage might be used to distinguish between SCZ patients and healthy controls.

### Correlation between clinical characteristics and LOY in the blood of SCZ patients

**Fig. 4:**
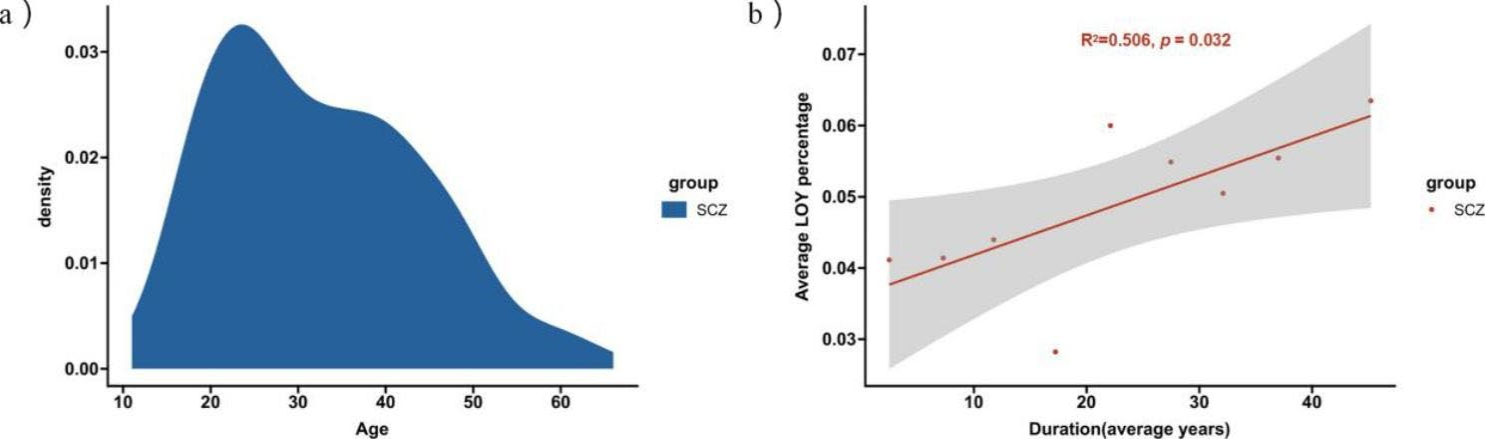
**(a)** Demonstrates the distribution of the age of onset of 186 patients with SCZ. The incidence is higher in early adulthood (20s), which is in accordance with the previous studies. The x-axis represents the age of onset. **(b)** shows the correlation between the duration of illness and the LOY percentage, where the value of x-axis at each point is the average of a certain duration range, and the value of y-axis at each point is the average LOY percentage within the corresponding range. The correlation index R^2^ is 0.506 with a *p*-value equal to 0.032

We determined the age of onset in 186 patients and found that the incidence peaked in their early 20s (Fig. 4a). This result is consistent with previous findings and supports the conclusion that SCZ usually occurs in early adulthood [15].

SCZ is usually recurrent and prolonged. Therefore, when exploring the relationship between disease duration and the LOY percentage, we tried to reduce errors by using the average duration and the average LOY percentage. The results showed that the LOY percentage increased significantly with the duration of illness (R^2^ = 0.506, *p* = 0.032) (Fig. 4b).

To further explore whether these factors contribute to the final LOY, we performed logistic regression analysis in which LOY was the response variable, and the age of onset and duration of illness were the explanatory variables (Table 2). The results were all statistically significant. This result suggests that the age at onset and the duration of disease were risk factors for LOY in the present study. Moreover, the result regarding the duration of the disease was consistent with the study in the Japanese population [30]. This implies that the risk of LOY increases by 0.058 and 0.057 per year with the age at onset and duration of disease, respectively (*p*_onset_ = 0.013, *p*_duration_ = 0.017).

**Table 2:** Logistic regression analysis of LOY in blood samples of patients with schizophrenia (n = 186)

Explanatory variable	Response variable: LOY [(X-Y)/X > 0.1]			
	B*	*P* - value	OR	95% CI
Age of Onset	0.056	**0.013**	1.058	1.012–1.105
Duration of SCZ	0.055	**0.017**	1.057	1.010–1.106

**Notes**: For logistic regression analyses, LOY was the response variable, while age of onset and duration of SCZ were the explanatory variables. Boldface type indicates statistical significance (P < 0.05). *B represents the unstandardized partial regression coefficient.

**Abbreviation**: LOY, loss of chromosome Y; SCZ, schizophrenia.

### Additional tests for samples with high LOY percentages

During the process, we observed several samples (sample IDs: 141, 291, and 325 in the schizophrenia group) with high LOY percentages; we validated these percentages using the Microreader™ 19X Direct ID System (Beijing Microreader Genetics, Beijing) based on capillary electrophoresis (CE). The profiles showed that these samples presented two alleles at certain X chromosome loci (Figure S2); thus, we speculate that these samples had the 47 XXY karyotype. This would explain the high LOY percentages. This also highlights that when outliers are detected, additional tests should be performed for detailed evaluation.

## Discussion

Research on the relationship between SCZ and LOY has provided insight and motivated the current study [30]. However, this study is the first conducted with the Han Chinese population and with a larger sample size. It was found in a previous study that robust and reproducible results via the SNP-array method could be obtained when the loss of chromosome Y occurred in > 10% of nucleated cells in blood samples [31, 32]. Therefore, we used both the LOY percentage and LOY presence (i.e., a binary variable, yes/no) to describe the phenomenon of LOY based on whether a set threshold was reached (LOY was considered present with a LOY percentage > 0.1). Additionally, this setup facilitated the comparison of our findings in the Han Chinese population with those in the Japanese population. According to the results, consistent with the healthy controls, patients exhibited a slight upwards trend in the LOY percentage with increasing age, but this trend was not significant (*p* = 0.081 in the SCZ group while *p* = 0.006 in the control group). Further examination by age group could increase the positive correlation. Moreover, in line with the findings in the Japanese population, the prevalence of LOY did not differ significantly between the two groups, but a significant difference in the LOY percentage between the SCZ group and the control group was found (*p* < 0.05). Thus, the LOY percentage has the potential to differentiate between SCZ patients and healthy individuals.

Currently, most data in LOY studies are derived from the total DNA of blood rather than the DNA of a particular type of white blood cell. Thus, we tested the total DNA of peripheral venous blood samples from the participants, which facilitated comparison and determination of the LOY characteristics of different disease groups. With the recent expansion of LOY research, the approaches used to measure LOY have been updated. In this study, we measured LOY by ddPCR, which was developed by Daniel et al. [25] for absolute quantification of the frequency of LOY. This differs from the technical approach used by Takashi Hirata et al. [30] in that it has higher sensitivity and precision, requires no standards, and applies to trace amounts of the template.

Consistent with previous studies, participants with SCZ in the present study were likely to have their first episode (SCZ onset) in their 20s. Our findings suggest that age of onset and duration of illness are risk factors for LOY and that the LOY percentage increased with disease duration. Notably, disease duration is inevitably associated with the natural ageing of the patient. As previously reported in most studies investigating the association between age and LOY [19, 23, 31, 33], the frequency of LOY in leukocytes itself might increase with age. We also examined whether there were differences in the LOY percentage between patients with different symptoms (**Figure S3**). There were significant differences in the LOY percentage between the negative-symptom group and the delusional group as well as between the negative-symptom group and the abnormal-speech-and-behaviour group. This seems to suggest a possible connection between the properties of symptoms and the LOY percentage, but it remains unclear which variable is the cause and which is the effect. Since symptoms within the same patient may vary with the stage of the illness, and multiple symptoms may be present, the present results are unable to fully elucidate this complex relationship. In addition, many factors, such as the type and dose of drugs used and smoking status, may also have an impact on the analysis of LOY. However, a more in-depth analysis is limited because of incomplete information. The sample size and variety should be expanded in future studies to enable assessment of the effect of a particular factor on LOY using control variables. Notably, specific and accurate clinical information and uniform assessment criteria are expected in future studies.

The results of previous studies on the association between smoking and LOY shed some light on our findings regarding the relationship between the stage of the convalescence of SCZ and LOY. Smoking status is an essential factor affecting LOY, as previously reported, and the percentage of LOY was associated with smoking status. Specifically, the frequency of LOY was higher in smokers than in never smokers, while no significant difference was found between ex-smokers and never smokers. This revealed a dose-dependent mutagenic effect of smoking on LOY risk [21, 34]. Since our results revealed no significant difference in LOY according to the presence of SCZ, we suspect that the status of LOY probably changed with the stage of the convalescence of SCZ, and thus might have influenced the results of the current study. However, this needs to be explored more deeply in the future.

Negative LOY percentages have also been observed, indicating a Y chromosome gain. This phenomenon was observed in a longitudinal study on LOY in peripheral blood samples from adult males in Uppsala [25]. As early as 1987, a study found an increase in the presence of the Y chromosome in one of 12 male lung cancer samples by the Southern blot hybridization method [35]. There is no explanation for such phenotypic results regarding the gain of chromosome Y (GOY); however, it may be related to the abnormal proliferation of specific cell types, the mechanisms of which need further investigation.

We conducted additional tests on samples with unusually high LOY percentages, and the 47, XXY chromosomal pattern found in this study suggests the presence of Klinefelter’s syndrome in these male participants; this condition might be involved in the aetiology of SCZ [36]. The effects of an extra X chromosome on cognition, particularly language impairments, were assessed in a previous study [37]. Interestingly, language impairments are also prominent in people with SCZ and might lead to other clinical symptoms, such as disorganization of language and thought [38, 39]. Moreover, there are candidate risk genes for SCZ on the X chromosome [40]. The increased copy numbers of these genes may be involved in the development of SCZ in individuals with X chromosome aneuploidies [41]. Although the mechanism by which the duplicated X chromosome influences schizophrenia is not clear, it has been reported that the risk of SCZ is almost four times higher in patients with Klinefelter’s syndrome [42]. It would be interesting to explore the incidence of 47, XXY karyotype in SCZ patients and healthy controls in future studies.

The Y chromosome is involved in many other biological processes in addition to sex determination and spermatogenesis. LOY is thought to be a potential biomarker of genomic damage and instability [31, 43], and there is growing evidence that genomic and epigenomic instability is associated with neuropsychiatric disorders aside from SCZ [44]. Thus, both the relationship between LOY and other psychiatric disorders and the pathophysiological mechanisms should be explored in future studies.

## Conclusion

The present study showed that in the Han Chinese population, SCZ patients have a slightly higher LOY percentage than healthy controls; this difference in the LOY percentage was significant and may be potential to distinguish between the two groups. However, there was no significant difference in the prevalence of LOY between the two groups. Nonetheless, increased age of onset and prolonged duration of illness appear to be risk factors for LOY. To better understand the relationship between LOY and SCZ, a larger cohort with more specific and accurate information is needed.

## Electronic supplementary material

Below is the link to the electronic supplementary material.

Supplementary Material 1

Supplementary Material 2

## Data Availability

All data generated or analysed during this study are included in this published article [and its supplementary information files].

## Abbreviations

AMEL: Amelogenin gene
SCZ: schizophrenia
WHO: the World Health Organization
CNVs: copy number variants
LOY: loss of chromosome Y
FISH: fluorescent in situ hybridization
SNP: single nucleotide polymorphism
ddPCR: droplet digital PCR
PSM: propensity score matching
IQR: interquartile range
GOY: gain of chromosome Y
CE: capillary electrophoresis
