# Supplementary material for: Characterization of loss of chromosome Y in peripheral blood cells in male Han Chinese patients with schizophrenia
Source: BMC Psychiatry. 2023 Jun 27;23:469. doi: 10.1186/s12888-023-04929-z (PMC10304327; doi:10.1186/s12888-023-04929-z)
Supplement: Supplementary file 2 — Supplementary Material 2 [file 12888_2023_4929_MOESM2_ESM.docx]

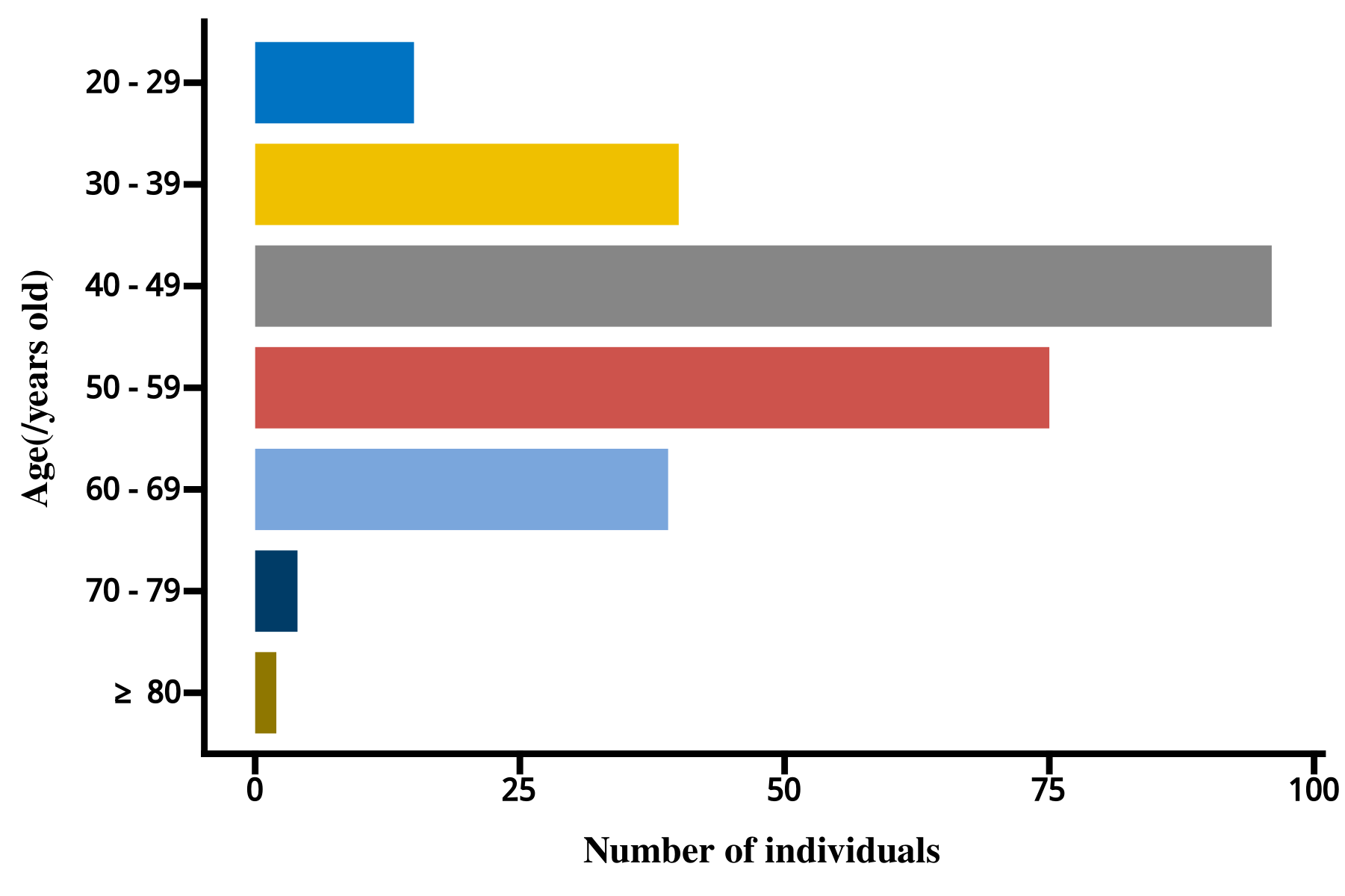


**Fig S1.** The age composition of the 271 patients with schizophrenia involved in this study. The x-axis indicates the number of individuals in relevant age group, and y-axis is clustered by each age group.


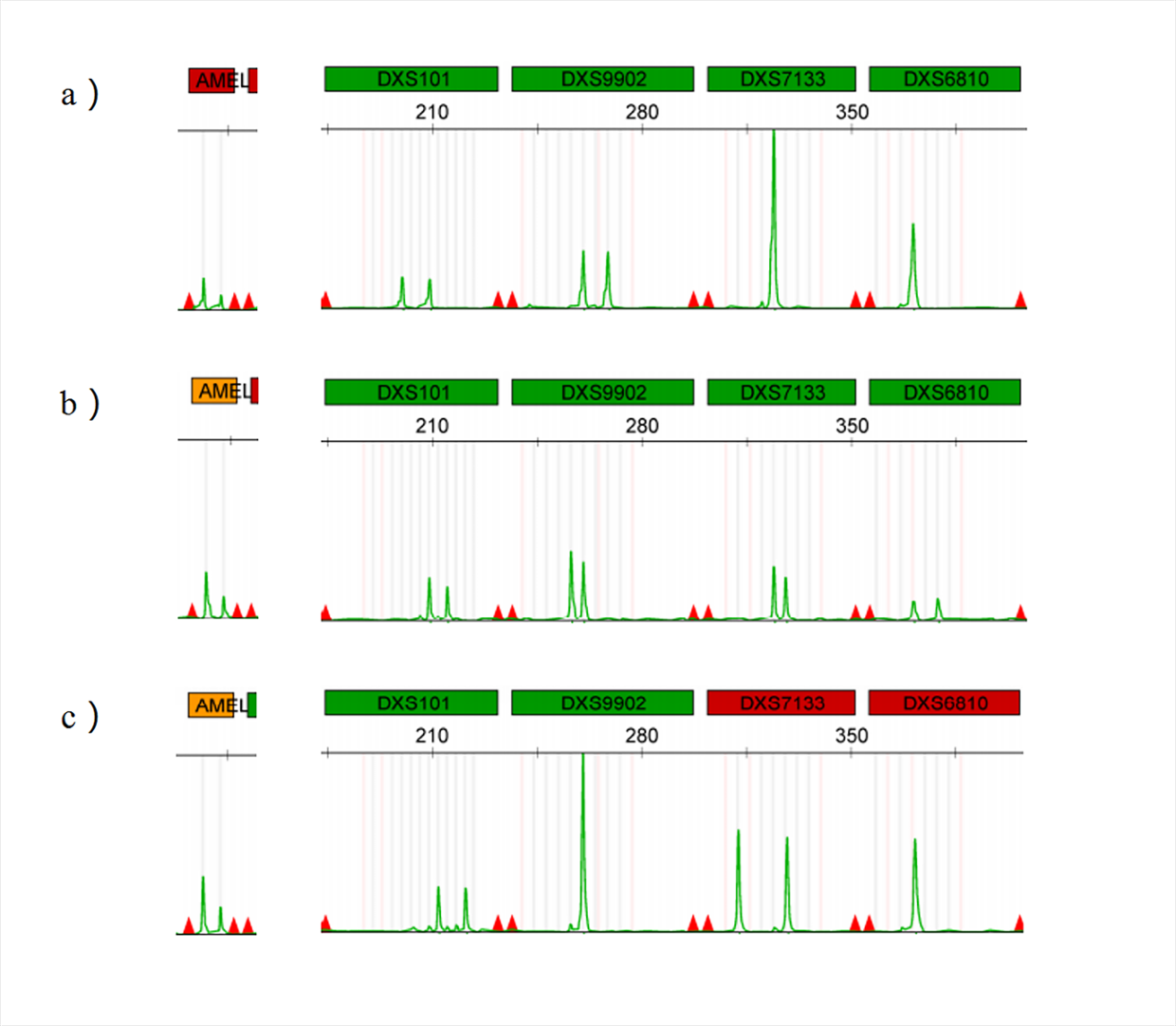


**Figure S2.** The partial profiles of three samples with high LOY percentage using the Microreader^TM^ 19X Direct ID System. a) is from the sample 325, b) is from the sample 291, and c) is from sample 141.


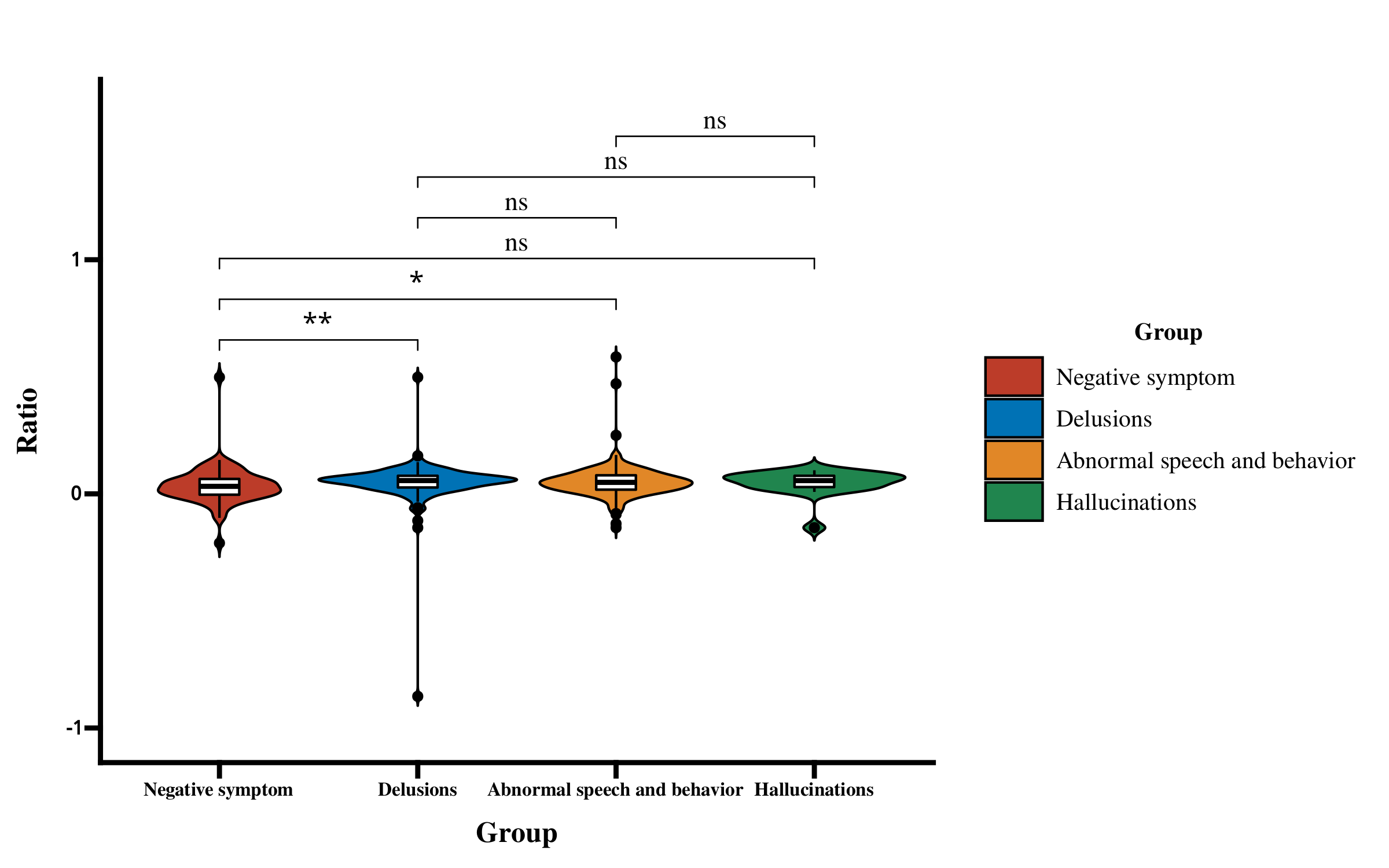


**Figure S3.** Differences in the LOY percentage (ratio) between groups after subgrouping schizophrenia patients according to typical symptoms, including negative-symptom, delusions, abnormal-speech-and-behavior, and hallucinations. The differences were very slight, while only the differences between the delusional and negative symptom groups and between the abnormal speech and behavior group and the negative symptom group were statistically significant.
